# Under-nutrition and associated factors among children infected with human immunodeficiency virus in sub-Saharan Africa: a systematic review and meta-analysis

**DOI:** 10.1186/s13690-021-00785-z

**Published:** 2022-01-05

**Authors:** Jemberu Nigussie, Bekahegn Girma, Alemayehu Molla, Moges Mareg, Esmelealem Mihretu

**Affiliations:** 1 Department of Nursing College of Health Sciences and Medicine, Dilla University, Dilla, Ethiopia; 2 Department of Psychiatry College of Health Science and Medicine, Dilla University, Dilla, Ethiopia; 3 Department of Reproductive Health School of Public Health, College of Health Science and Medicine, Dilla University, Dilla, Ethiopia; 4 Department of Nursing College of Health Sciences and Medicine, Debre Markos University, Debre Markos, Ethiopia

**Keywords:** Under-nutrition, Malnutrition, Children, Sub-Saharan Africa, Human immunodeficiency virus, HIV positive children

## Abstract

**Background:**

In the developing world, such as the sub-Saharan African region, HIV/AIDS has worsened the impact of under-nutrition in children. HIV infected children are highly vulnerable to under-nutrition. Therefore, the objective of this systematic review and meta-analysis was to estimate the pooled prevalence of under-nutrition, and the pooled effect sizes of associated factors among HIV-infected children in sub-Saharan Africa.

**Methods:**

The primary studies for this review were retrieved from PubMed/ MEDLINE online, Science Direct, Hinari, web of science, CINHAL, EMBASE, WHO databases, Google, and Google Scholar databases. The articles selected for this meta-analysis were published between 2010 and 2020. The last search date was 18 October 2021. The data was extracted in Microsoft Excel format and exported to STATA Version 14.0. A random effect meta-analysis model was used. Heterogeneity was evaluated by the I^2^ test. The Egger weighted regression test was used to assess publication bias.

**Results:**

We retrieved 847 records from these databases. Of which records, 813 were excluded due to different reasons and 34 studies were included in the final analysis. The pooled prevalence of stunting, underweight and wasting in HIV infected children was 46.7% (95% CI; 40.36–53.07, I^2^ = 98.7%, *p* < 0.01), 35.9% (95% CI; 30.79–41.02, I^2^ = 97.4% *p* < 0.01), and 23.0% (95% CI; 18.67–27.42, I^2^ = 96.9%, *p* < 0.01) respectively. The advanced WHO HIV/AIDS clinical staging (III&IV) [OR = 6.74 (95%: 1.747, 26.021), I^2^ = 94.7%] and household food insecurity were associated with stunting [OR = 5.92 (95% CI 3.9, 8.87), I^2^ = 55.7%]. Low family economic status [OR = 4.737 (95% CI: 2.605, 8.614), I^2^ = 31.2%] and increased feeding frequency [OR = 0.323 (95% CI: 0.172, 0.605), I^2^ = 69.8%] were significantly associated with under-weight. Anemia [OR = 2.860 (95% CI: 1.636, 5.000), I^2^ = 74.8%] and diarrhea in the previous month [OR = 4.117 (95% CI: 2.876, 5.894), I^2^ = 0.0%] were also associated with wasting among HIV infected children in sub-Saharan Africa.

**Conclusions:**

The pooled prevalence of under-nutrition among HIV infected children was high. Nutritional assessment and interventions need great attention as a part of HIV care for HIV positive children. The implementation of policies and strategies established by national and international stakeholders in ART care centres should take a maximum emphasis on reducing under-nutrition among HIV infected children.

**Supplementary Information:**

The online version contains supplementary material available at 10.1186/s13690-021-00785-z.

## Background

Worldwide, both under-nutrition and human immunodeficiency virus (HIV) are highly prevalent, particularly in the sub-Saharan Africa region [1]. In the world, nearly 2.84 million children under 19 years in 2019 were living with HIV, and more than 90% were in the sub-Saharan Africa region [2]. In 2018, approximately 49 and 149 million under-five children were stunted and wasted, respectively, and more than 90% lived in low and middle-income countries [3]. The magnitude of stunting and wasting in Sub-Saharan Africa varies in the region by as much as 32 and 10%, respectively [4]. HIV/AIDS, poverty, and food insecurity were the main causes of these high under-nutrition problems [5]. Studies have shown that stunting, under-weight, and wasting were more prevalent among HIV infected children than uninfected children [6–8].

Under-nutrition is responsible for about 11% of the global disease burden [9], more than 35% of child deaths [10], and deformities such as cognitive impairment, chronic diseases, and growth failure [10]. In a resource-limited setting, more than one-third of under five children mortality was due to under-nutrition every year [11]. In HIV infected children the risk of death due to under-nutrition is three-times higher than non-HIV infected children [12]. The magnitude of severe under-nutrition, hospitalization and death rates reaches as high as 20–50% among HIV infected or exposed children in sub-Saharan Africa [13, 14].

HIV/AIDS, under-nutrition, and lack of essential micronutrients affect the immune system, leading to a nutritionally acquired immune dysfunction syndrome that increases susceptibility to infection which complicates the case managements [13, 15, 16]. HIV infection increases the risk of under-nutrition due to the high activity of pro-inflammatory cytokines that causes growth impairment [17]. HIV-related opportunistic infections, such as persistent diarrhoea, oral, and oesophageal candidiasis, have negative impact on the nutritional status of the patient [18]. Furthermore, initially ART in children can also cause metabolic disorders and adverse effects on nutritional status that causes complications such as nausea and vomiting or reduced bone mineral density, especially in the first months of treatment [19].

The clinical context and interventions for most causes of childhood mortality worldwide have been addressed over the last decade [18, 20], but the management of under-nutrition in children, particularly those infected with HIV, remains poorly addressed [21]. Studies reported differing magnitudes of low nutritional status of HIV infected children and identified study setting-specific factors. The prevalence of stunting, under-weight and wasting in sub-Saharan Africa ranged from 13.4 to 77%, 6.8 to 56.3% and 2.5 to 52% respectively [22–27]. This showed pronounced discrepancies among reports of under nutrition across different geographical settings and different time periods. Furthermore, there are no regionally represented pooled data of under nutrition in in sub-Saharan Africa.

Subsequently, reliable and summarized information is essential to refine governments’ policies, strategies, and interventions. Therefore, the aim of this systematic review and meta-analysis was to estimate the pooled prevalence of under-nutrition, and the pooled effect sizes of factors associated with under-nutrition among HIV infected children in sub-Saharan Africa. Therefore, this review can be of vital importance in showing summarized evidence and suggesting possible applicable strategies for planning, decision making, and resource allocation in the health care system of the sub-Saharan Africa region.

### Review question

What is the pooled prevalence of under-weight, wasting and stunting among HIV infected children in the sub-Saharan Africa from 2010 to 2021?

What is the pooled effect size of associated factors for under-nutrition among HIV infected children in the sub-Saharan Africa from 2010 to 2021?

## Methods

### Study identification

The Preferred Reporting Items for Systematic Reviews and Meta-Analyses (PRISMA) guideline was used to write this systematic review and meta-analysis [28]. The published and unpublished literature (Grey literature) describing the prevalence and associated factors of under-nutrition (stunting, wasting, and under-weight) among HIV infected children were reviewed.

### Eligibility criteria

Observational studies including cross-sectional, comparative cross-sectional, case-control, and cohort studies reporting the prevalence of under-nutrition among children infected with HIV in sub-Saran Africa published from 2010 to 2021 were included for the first objective. Similarly, studies that identify factors associated with stunting, wasting or under-weight in the respective area published from 2010 to 2021 were included to estimate the pooled effect size of associated factors. In this review, we include articles published in English. Studies that didn’t reported neither the prevalence nor associated factors of under-nutrition (stunting, under-weight and wasting) were excluded. Case reports, qualitative studies, and articles without full text were not included in this systematic review and meta-analysis.

### Outcomes of measurement

This study has two main outcomes. The first result was the pooled prevalence of under-nutrition among HIV infected children in sub-Saharan Africa. The second outcome was to identify factors associated with stunting, underweight, and wasting among HIV infected children. In the included studies, the screening for stunting, under-weight, and wasting was performed by height for age- Z score (HAZ), weight for age -Z score (WAZ), and weight for height -Z score (WHZ), respectively. The prevalence was measured using the percentage of under-nutrition (stunting, under-weight, and wasting) among HIV positive children. The associated factors with stunting, underweight and wasting were measured in terms of the odds ratio. The odds ratio was calculated from primary studies using two- by- two epidemiological tables.

### Search strategy

Relevant studies were searched from the PubMed / MEDLINE online, Science Direct Hinari web of science, CINHAL, EMBASE, WHO databases. Grey literature was also identified from Google and Google Scholar. The key terms used to retrieve primary studies were Prevalence OR Magnitude AND Under-nutrition OR, Stunting OR Under-weight OR Wasting OR Malnutrition OR Nutritional status AND Human Immunodeficiency Virus (HIV) AND Children OR ‘child’ OR ‘infant’ AND Sub-Saharan Africa) for the first objective. We used key terms ((Factors OR determinants OR risk factors OR correlates) AND Under-nutrition OR Under-weight OR Wasting OR Stunting /Malnutrition/ AND human immunodeficiency virus (HIV) AND Children AND Sub-Sahara Africa) to search primary studies conducted on factors associated with under-nutrition among HIV infected children (supplementary file five). The last search date was 18 October 2021. The search of the studies was done by JN and BG.

### Quality appraisal

The principal investigator (JN) performed an initial review by title and abstract to eliminate articles that were visibly not important for this review. The full text articles were included if they reported the magnitude or prevalence of under-nutrition (stunting, under-weight or stunting) and/or its associated factors. Two investigators (BG and AM) independently screened the selected full text studies using our eligibility criteria. During the selection process, disagreements between the two authors were resolved by mediation of the fourth reviewer (MM) for the final decision to be included in the analysis.

The quality of the included studies was assessed using the Newcastle-Ottawa quality assessment scale [29]. The tool has three main parts. The first part had five components used to assess the methodological quality of each study. The second part assesses the comparability of primary studies, and the final part of the tool measures the quality of the original articles with respect to their outcome and statistical analysis. Two authors (JN, BG) independently assessed the methodological quality, the quality of the reported data, the stratified data on the types of patients (stunted, under-weight, and wasted), and the clarity of the research design of the included study. Any difference between the two authors during the quality assessment of the primary studies was resolved by taking the average of the two assessment scores. Articles scored 7 and more can be considered as low risk and good to be included for the meta-analysis.

### Data extraction

We used a standardized data extraction format prepared in Microsoft Excel for each type of under-nutrition (stunting, under-weight, wasting) extract all the necessary data. The extraction format contains the name of the first author, publication year, country where the study was conducted, sample size, outcome, response rate, study design, and prevalence of stunting, underweight, wasting for the first objective. For the second objective (factors associated with stunting, underweight, wasting), the Microsoft Excel data extraction format was prepared in the form of a two –by- two table. Categorical variables were tabulated (a, b, c and d) with the outcome variable (stunting, underweight, and wasting) during extraction. Data were extracted by two authors (JN, BG) using a standardized data extraction spread sheet. The third and fourth authors (AM, MM) assessed the accuracy of the extracted data.

### Data analysis and interpretation

The data extracted from the Microsoft Excel format were exported to STATA Version 14.0 (software) for analysis. A random effect meta-analysis model was used. The pooled effect size was employed in the form of prevalence and odds ratio for all type of under-nutrition (stunting, wasting and under-weight). The Forest plot was used to show the pooled estimate with a 95% confidence interval (CI). Statistical heterogeneity was evaluated by the I^2^ test, which shows the level of heterogeneity between studies [30]. Basically, the I^2^ test doesn’t depend on the number of studies incorporated into the study. The heterogeneity of the included studies was interpreted as an I^2^ value of 25% = low, 50% = moderate, and 75% and above = high. We also assessed publication bias by visual inspection of funnel plots. To identify the source of heterogeneity, sub-group analysis was performed using country, study design, and year of publication as criteria. Egger’s weighted regression test was used to assess publication bias at the 5% significant level [31, 32]. Finally, for all analyses, *P* < 0.05 was considered statistically significant.

## Result

**Fig. 1:**
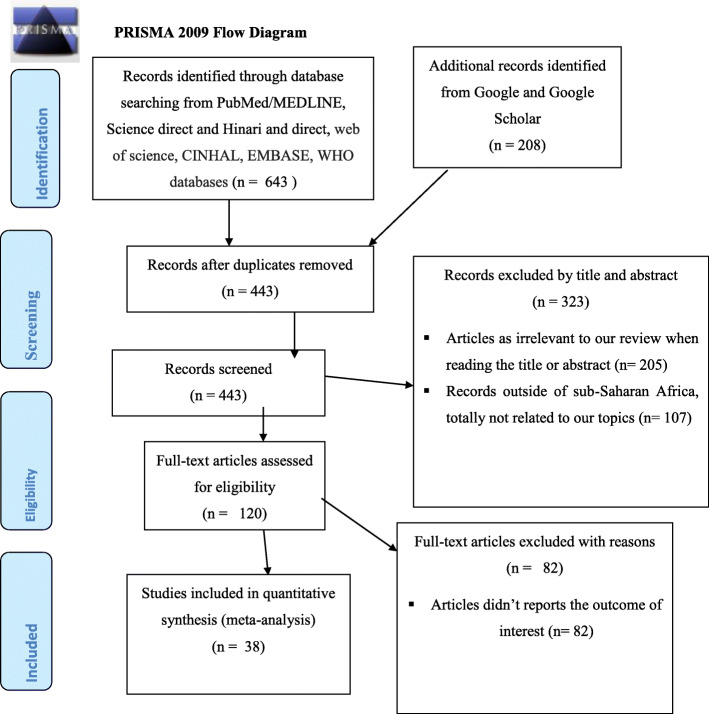
PRISMA flow diagram of included studies to estimate the pooled prevalence of under-nutrition among HIV infected children in Sub-Saharan Africa, 2021

In the initial search, we found a total of 1034 records from the electronic search database of Midline/PubMed, Science Direct, Hinari, web of science, CINHAL, EMBASE, WHO databases Google, and Google Scholar. About 591 records were excluded due to duplication, and the remaining 443 records were screened. About 323 articles were excluded after reading their title and abstract as we found these articles irrelevant to our review. After the full text review, 82 articles were further excluded with reason. Finally, 38 studies were included in this systematic review and meta-analysis **(**Fig. 1).

### Characteristics of the included articles

This systematic review and meta-analysis included 38 primary articles with a total of 16,790 study participants published between 2010 and 2021 from more than 16 sub-Saharan African countries [6–8, 22–27, 33–61] (Table 1). All primary studies included in this systematic review and meta-analysis were observational studies conducted in health facilities with a sample size 28 to 3195 from study conducted in South Africa [48] and West Africa [46] respectively. All included studies used WAZ, WHZ, and HAZ scores below − 2 Z- scores (WHO standard) to screen under-weight, wasting, and stunting, respectively.

**Table 1:** Distribution of included studies on the prevalence of under-nutrition among HIV infected children Sub-Saharan Africa, 2021

Author	Publication year	Country	Sample size	Stunting (%)	Under-weight (%)	Wasting (%)	Study design	reference
Kusum Lata et al	2020	Ethiopia	420	60.20	41.20	21.40	Cross-sectional	[33]
Sunguya et al	2011	Tanzania	213	36.60	22.10	13.60	Cross-sectional	[6]
Henry Chineme et al	2014	Nigeria:	70	48.60	58.60	31.40	Cross-sectional	[7]
Maura Pedrini et al	2015	Mozambique	140	57.40	47.10	18.60	Cross-sectional	[34]
Jesson et al	2015	Central and west Africa	1350	32.90	36.00	16.50	Cross-sectional	[35]
Megabiaw et al	2012	Ethiopia	301	65.00	41.70	5.80	Cross-sectional	[36]
Poda et al	2017	Burkina faso	164	29.90	11.60	10.40	Cross-sectional	[8]
Calixte Ida Penda et al	2018	Cameroon	217	63.60	37.80	18.40	Cohort	[37]
Bruno F. Sunguya et al	2014	Tanzania	748	61.90	26.50	6.30	Cross-sectional	[38]
Andreas Chiabi et al	2012	Cameroon	39	51.30	56.40	20.50	Cohort	[39]
A.F. Fagbamigbe et al	2019	Nigeria:	390	36.00	50.00	50.00	Cross-sectional	[40]
E. A. anigilaje et al	2015	Nigeria:	180	54.40	12.10	33.50	Cross-sectional	[41]
Teklemariam et al	2015	Ethiopia	108	49.10	51.60	31.50	Cross-sectional	[42]
R. S. Mwiru et al	2014	Tanzania	3144	52.00	40.00	30.00	Cohort	[43]
Jesson J et al	2018	West Africa	161	52.00	52.00	36.00	Cross-sectional	[44]
Cames et al	2017	Senegalese	244	42.00		52.00	Cross-sectional	[27]
Ute D. Feucht et al	2016	South Africa	159	73.00	50.00	19.00	Cohort	[45]
Julie Jesson. et al	2019	West Africa	3195	50.20	55.70	39.70	Cohort	[46]
Sofeu CL et al	2019	Cameroon	210	77.00	53.00	47.60	Cross-sectional	[23]
McHenry MS. et al	2019	Kenya	426	50.90	26.50	13.60	Cohort	[47]
Kimani-Murage et al	2011	South Africa	28	28.60	10.70	7.00	Cross-sectional	[48]
Sunguya et al	2012	Tanzania	219	40.10	6.80	10.00	Cross-sectional	[24]
R. Weigel et al	2010	Malawi	363	69.10	51.80		Cohort	[49]
Tekleab et al	2016	Ethiopia	202	71.30	39.50	16.30	Cohort	[50]
David Aguilera et al	2019	Equatorial guinea	213	56.30	56.30	27.70	Cross-sectional	[25]
Julie Jesson et al	2017	Mali	308	20.00		31.50	Cohort	[51]
Asiya et.al.	2018	Ethiopia	412	13.40	21.80		Cross-sectional	[22]
Haileselassie et al	2019	Ethiopia	376	24.70		28.20	Cross-sectional	[52]
Arinaitwe et al	2012	Uganda	57	29.89	29.89		Cohort	[53]
Atnafu Mekonnen et al	2014	Ethiopia	243	62.10	15.4	2.50	Cross-sectional	[26]
Kedir et al	2014	Ethiopia	560		51.6		Cohort	[54]
Abdulkadir et al	2014	Ethiopia	142	46.50	40.80	31.70	Cross-sectional	[55]
Arpadi et al	2019	Rwanda	374	60.00	24.00	11.00	Cross-sectional	[56]
Nalwoga et al	2010	Uganda	31	68.00	52.00	4.00	Cross-sectional	[57]
S. T. Echendu et al	2021	Nigeria	370	27.9	15.7	13.3	Cross-sectional	[60]
Dessalegn N. et al	2021	Ethiopia	360	30.3	19.4	19.2	Cross-sectional	[59]
Shiferaw and Gebremedhin	2020	Ethiopia	260	33.1		20.0	Cross-sectional	[58]
Tiruneh et al	2021	Ethiopia	393	5.5		36.3	Cross-sectional	[61]

The highest prevalence of stunting was reported from a study conducted in Cameron (77.0%) [23], and the lowest was reported from a study conducted in Ethiopia (5.5%) [61]. Similarly, the highest prevalence of underweight was reported from a study in Nigeria (58.6%) [7], and the lowest was from a study in Tanzania (6.8%) [24]. The highest (52.0%) and lowest (4%) prevalence of wasting were also reported from study conducted in Senegalese [27] and Uganda [53] respectively.

### Meta-analysis

**Fig. 2:**
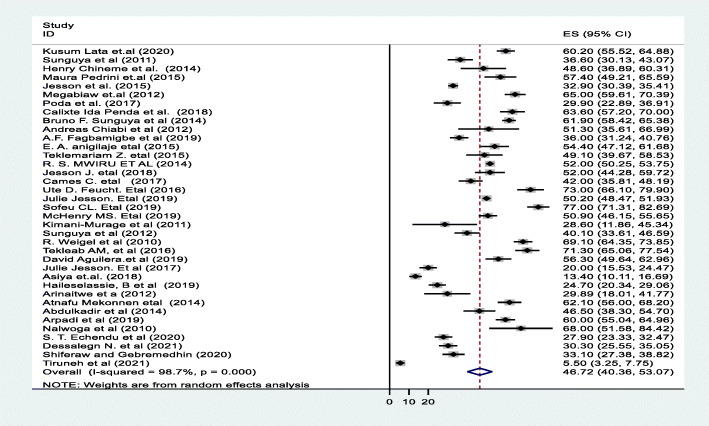
Forest plot for the pooled prevalence of stunting among HIV infected children in Sub-Saharan Africa, 2021

**Fig. 3:**
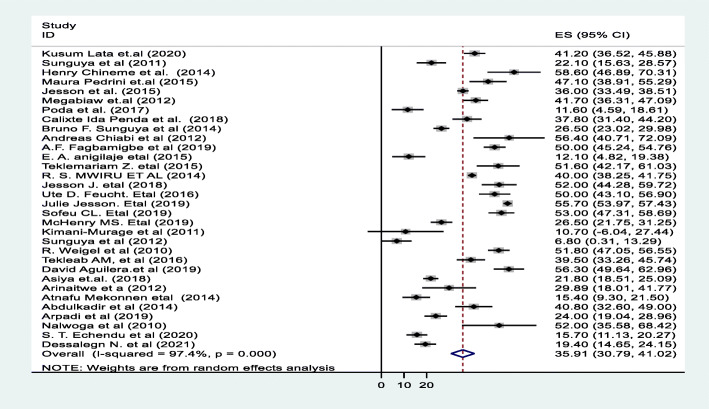
Forest plot for the pooled prevalence of under-weight among HIV infected children in Sub-Saharan Africa, 2021

**Fig. 4:**
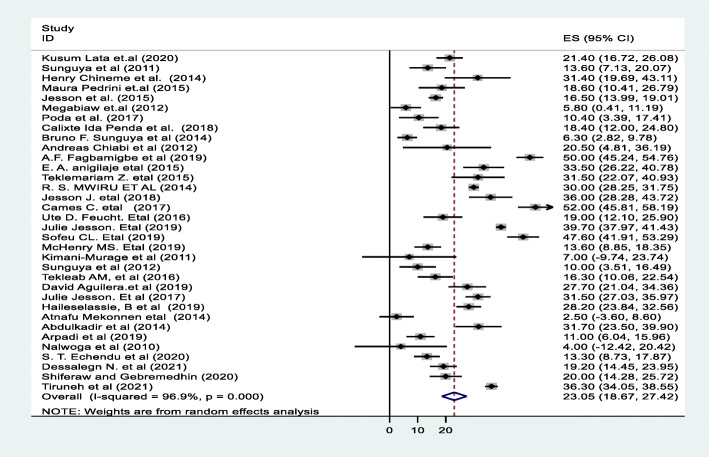
Forest plot for the pooled prevalence of wasting among HIV infected children in Sub-Saharan Africa, 2021

**Fig. 5:**
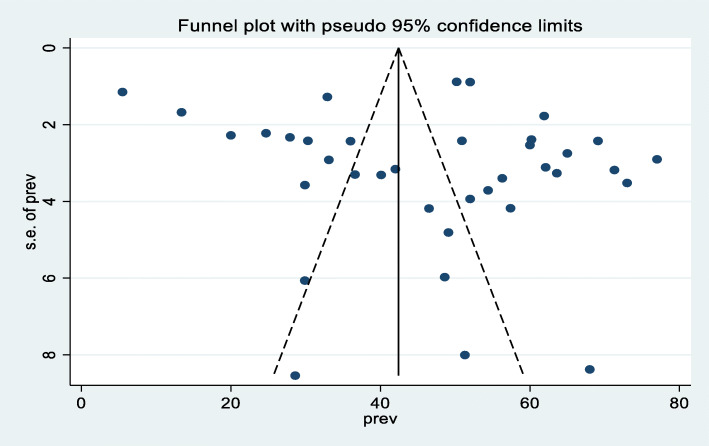
Funnel plot showing the symmetric distribution of articles on pooled prevalence stunting among HIV infected children in Sub-Saharan Africa, 2021

**Fig. 6:**
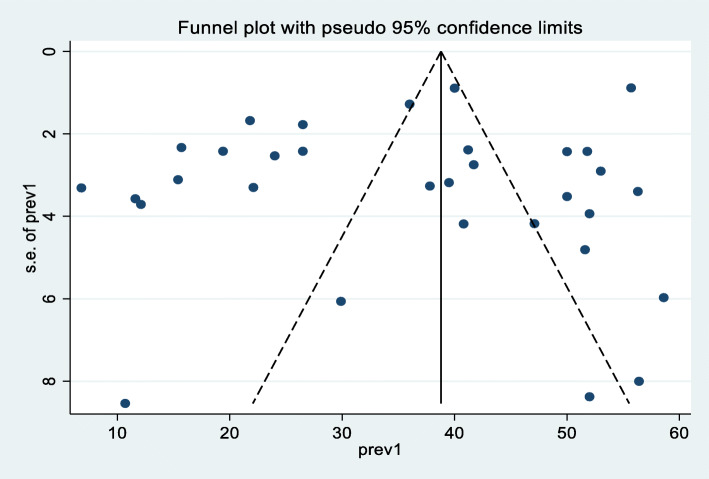
Funnel plot showing the symmetric distribution of articles on pooled prevalence of under-weight among HIV infected children in Sub-Saharan Africa, 2021

**Fig. 7:**
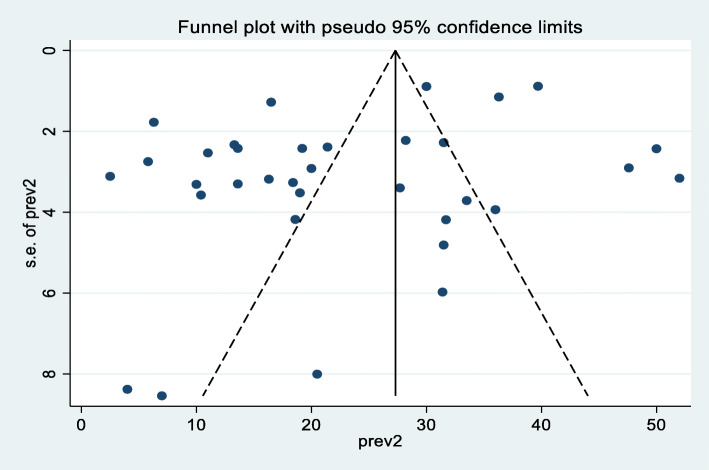
Funnel plot showing the symmetric distribution of articles on pooled prevalence of wasting among HIV infected children in Sub-Saharan Africa, 2021

A random effect meta-analysis model was used to estimate the pooled prevalence of under-nutrition and its associated factors among HIV infected children in sub-Saharan Africa. To estimate the prevalence of stunting, 37 studies were included in the analysis; the overall pooled prevalence of stunting was 46.7% (95% CI; 40.36–53.07, I2 = 98.7%, *p* < 0.01), (Fig. 2). Similarly to estimate the prevalence of underweight, 33 studies were included in the analysis, and the total pooled prevalence of under-weight was 35.9% (95% CI; 30.79–41.02, I^2^ = 97.4% *p* < 0.01), **(**Fig. 3**).** Thirty (34) studies were also included in the analysis to estimate the pooled prevalence of wasting among HIV-infected children; the overall pooled prevalence of wasting was 23.0% (95% CI; 18.67–27.42, I^2^ = 96.9%, *p* < 0.01) **(**Fig. 4**).** High heterogeneity was observed between studies on the prevalence estimate of stunting, under-weight and wasting. Publication bias was checked using the Egger’s test and the results showed that there was no significant publication bias, as evidenced by *p* = 0.285, 0.128 and 0.058for stunting, under-weight, and wasting, respectively. We also observed the symmetrical distribution of the funnel plots indicating that publication bias was not significant problem in this meta-analysis (Figs. 5, 6 & 7).

Subgroup analysis was performed using country, study design, and year of publication to identify the source of heterogeneity.

#### Subgroup analysis by country

The highest pooled prevalence of stunting among HIV infected children was in Cameroon, 65.6% ((95% CI: 52.8–78.3), I^2^ = 86.7%, *P* < 0.01) and the lowest was in Nigeria, 41.18% ((95% CI: 29.60–25.77), 92.7, < 0.01). The highest pooled prevalence estimate of under-weight was in Cameroon, 48.2% ((95% CI: 36.1–60.3), I^2^ = 85.3%)) and the lowest was in Tanzania, 24.1% ((95% CI; 10.9–37.3), I^2^ = 97.9%, *P* < 0.01). Similarly, the highest pooled prevalence of wasting was in Nigeria 32.05% ((95% CI: 12.49–51.61), 97.5, *P* < 0.01) and the lowest was in Tanzania, 15.1% (95% CI: 0.52, 29.62), I^2^ = 98.3%, *P* < 0.01) **(**Table 2).

**Table 2:** Summary of subgroup analysis for the prevalence of stunting, under-weight and wasting among HIV infected children in Sub-Saharan Africa, 2021

Type	Feature	Pooled prevalence of stunting, %(95% CI, I^2^, *P* value)	Pooled prevalence of under-weight, % (95% CI, I^2^, P value)	Pooled prevalence of wasting, %(95% CI, I^2^, P value)
Sub-group analysis by country	Ethiopia	41.83 (26.83–56.84, 99.1, < 0.01)	33.62 (25.04–42.20, 95.0, < 0.01)	21.24 (13.56–28.93, 95.9, < 0.01)
	Tanzania	48.10 (39.05–57.15, 95.4, < 0.01)	24.11 (10.88–37.33, 97.9, < 0.01)	15.07 (0.52–29.62, 98.3, < 0.01)
	Nigeria	41.18 (29.60–25.77, 92.7, < 0.01)	33.83 (11.81–55.85, 98, < 0.01)	32.05 (12.49–51.61, 97.5, < 0.01)
	Mozambique*	57.40 (49.21–65.59, −)	47.10 (38.91–55.29, −)	18.60 (10.41–26.79, −)
	Central Africa*	32.90 (30.39–35.40, −)	36.00 (33.49–38.51, −)	16.50 (13.99–19.00, −)
	Burkina Faso*	29.90 (22.89–36.90, −)	11.60 (4.59–18.61, −)	10.40 (3.39–17.41, −)
	Cameroon	65.56 (52.82–78.29, 86.7, < 0.01)	48.16 (36.06–60.26, 85.3, < 0.01)	29.24 (7.00–51.47, 95.8, < 0.01)
	Senegalese*	42.00 (35.80–48.19, −)	–	52.00 (45.81–58.19, −)
	South Africa	51.48 (7.99–94.97, 95.7, < 0.01)	31.12 (7.36–69.60, 94.5, < 0.01)	15.52 (4.85–26.19, 40.7, 0.194)
	Kenya*	50.90 (46.15–55.64, −)	26.50 (21.75–31.25, −)	13.60 (8.85–18.35, −)
	Malawi*	69.10 (64.35–73.85, −)	51.80 (47.05–56.55, −)	–
	Equatorial Guinea*	56.30 (49.64–62.96, −)	56.30 (49.64–62.96, −)	27.70 (21.04–34.36, −)
	Mali*	20.00 (15.53–24.47, −)	–	31.50 (27.03–35.97, −)
	Uganda	48.51 (11.17–85.84, 92.6, < 0.01)	40.19 (18.57–61.80, 78.1, < 0.01)	4.00 (12.42–20.42, −)
	Rwanda*	60.00 (55.03–64.96, −)	24.0 (19.04–28.97,-)	11.00 (6.04–5.97, −)
Sub-group analysis by study design	Cross-sectional	49.8 (42.5–57.0, 97.8)	35.29 (29.70–40.87, 95.9)	22.15 (16.29–28.00, 96.1)
	Cohort	48.7 (39.7–57.8, 97.6)	44.89 (35.93–53.86, 97.2)	27.55 (20.99–34.11, 95.3)
Sub-group analysis by publication year	January 2010-december 2015	50.48 (44.19–56.77, 95.9,< 0.01)	34.82 (28.99–40.65, 95.1, < 0.01)	17.67 (11.45–23.89, 95.3, < 0.01)
	January 2016-aguest 2021	43.79 (33.998–53.575, 99.1, < 0.01)	36.93 (28.03–45.83, 98.3,< 0.01)	26.97 (21.37–32.58, 96.8, < 0.01)

*Countries having single study

#### Subgroup analysis by study design

The prevalence of stunting among HIV infected children was found to be 49.8% ((95% CI: 42.5–57.0, I^2^ = 97.8, *P* < 0.01) in cross-sectional studies and 48.7% (95% CI; 39.7–57.8), I^2^ = 97.6, *P* < 0.01) in cohort studies. The pooled prevalence of under-weight in cross-sectional studies was 35.3% (95% CI; 29.70–40.87, I^2^ = 95.9%, *P* < 0.01) while in cohort study it was found to be 44.9% (95% CI; 35.9–53.7, I^2^ = 97.2%, *P* < 0.01). The prevalence of wasting among HIV infected children was found to be 22.1% (95% CI; 16.3–28.0, I^2^ = 96.1, *P* < 0.01) in cross-sectional studies and 27.6% (95% CI; 20.99–34.11, I^2^ = 95.3%, *P* < 0.01) in cohort studies Table 2**).**

#### Sub-group analysis by year of publication

The pooled prevalence of stunting among HIV infected children was found to be 50.5% ((95% CI; 44.2–56.8), I^2^ = 95.9%, *P* < 0.01) from studies published from January 2010–December 2015, but it was 43.79% (95% CI; 33.998–53.575), I^2^ = 99.1%, *P* < 0.01) from studies published from January 2016–August 2021. The pooled prevalence of under-weight among HIV infected children was found to be 34.8% (95% CI; 28.99–40.65, I^2^ = 95.1%, *P* < 0.01) from studies published from January 2010–December 2015, while it was 36.9% (95% CI; 28.03–45.83, I^2^ = 98.3%, *P* < 0.01) from studies published from January 2016–August 2021. The pooled prevalence of wasting from studies published from January 2010 to December 2015 was found to be 17.7% (95% CI; 11.5–23.9, I^2^ = 95.3%, *P* < 0.01) while it was 26.97% (95% CI; 21.37–32.58, I^2^ = 96.8%, *P* < 0.01) from studies published from 2016 to 2021 **(**Table 2**).**

### Factors associated with under-nutrition among HIV infected children

#### Factors associated with stunting

During the review process, we identified numerous factors associated with stunting from the primary studies. Variables reported as a significant association with stunting in at least three primary studies were included in this metal analysis. Accordingly, advanced WHO HIV/AIDS clinical staging and household food insecurity were found to be a significant association with stunting (Table 3, supplementary file three).

**Table 3:** Summary of factors associated with under-nutrition among HIV infected children in Sub-Saharan Africa, 2021

Types of Under-nutrition	Variables	Number of studies	Studies includes the analysis	Odds ratio with 95%CI	Heterogeneity	
					(I^**2**^)	P- value
Stunting	WHO HIV/AIDS clinical staging	3	Haileselassie, B et al., 2019 Sunguya et al., 2011 Bruno F. Sunguya et al., 2014	6.74 (1.75, 26.02),	94.7%	*P* < 0.01
	Household food insecurity	4	Haileselassie, B et al.,2019 Sunguya et al., 2011 Bruno F. Sunguya et al., 2011 Sunguya et al., 2012	5.92 (3.9, 8.87)	55.7%	*P* = 0.079
Under-weight	low family income	4	Kusum Lata et.al, 2020 Megabiaw et.al, 2012 Sunguya et al., 2012 Asiya et.al, 2018	4.74(2.6, 8.61)	31.2%	*P* = 0.225
	Feeding frequency	3	Kusum Lata et.al, 2020 Sunguya et al., 2011 Bruno F. Sunguya et al., 2014	0.32 (0.17, 0.6)	69.8%	*P* = 0.037
Wasting	Diarrhoea	3	Kusum Lata et.al, 2020 Sunguya et al. 2011 Haileselassie, B et al., 2019	4.12 (2.88, 5.89)	0.0%	*P* = 0.386
	Anemia	3	Haileselassie, B et al., 2019 R. S. MWIRU ET AL 2014 Julie Jesson. Et al 2017	2.86 (1.64, 5.0)	74.8%	*P* = 0.019

Advanced WHO HIV/AIDS clinical staging was reported as a factor associated with stunting among HIV-infected children by three primary studies [6, 38, 52]. A total of 1337 participants were included to analyze the association between WHO HIV/AIDS clinical staging (III&IV) and stunting among HIV infected children. The pooled odds ratio showed that children who had an advanced WHO HIV/AIDS clinical stage were 6.74 times more odds of stunting than their counterpart [OR = 6.74 (95%: 1.747, 26.021), I^2^ = 94.7%, *P* < 0.01] (Table 3, supplementary file three).

Household food insecurity was reported to be a factor associated with stunting by four primary studies included in this review [6, 24, 38, 52]. A total of 1556 children were included to analyze the association between household food insecurity and stunting among HIV infected children. The pooled odds ratio showed that children in food-insecure households were 5.92 times more likely to develop stunting than children in food-secure households [OR = 5.92 (95% CI 3.9 -, 8.87), I^2^ = 55.7%, *P* = 0.079] (Table 3, supplementary file three)**.**

#### Factors associated with under-weight

To identify factors associated with under-weight, we reviewed more than 13 primary studies and identified numerous factors for the occurrence of under-weight among HIV infected children**.** Variables reported as a significant association with under-weight in at least three primary studies were included in this meta-analysis. As a result, low family income and feeding frequency were significantly associated with under-weight (Table 3, supplementary file three).

Family economic status was identified as a factor associated with underweight in four primary articles included in this meta-analysis [22, 24, 33, 36]. A total of 1352 participants were included to analyze the association between monthly family income and under-weight. The odds of under-weight among HIV infected children with low family income were 4.74 times higher than their counterparts [OR = 4.737 (95% CI: 2.605, 8.614), I^2^ = 31.2%, *P* = 0.225] (Table 3, supplementary file three).

Feed frequency was identified as a factor associated with under-weight among HIV infected children in three primary studies included in the meta-analysis [6, 33, 38] with a total of 1, 381 study participants. The odds of under-weight among HIV infected children who feed 4 times or more per 24 h were 67.7% less odds of under-weight than children feeding less than 4 times per 24 h [OR = 0.323 (95% CI: 0.172, 0.605), I^2^ = 69.8%, *P* = 0.037] (Table 3, supplementary file three).

#### Factors associated with wasting

In this review, we find numerous factors associated with wasting reported in different primary studies. Variables reported as a significant association with wasting in at least three primary studies were included in this metal analysis. Accordingly, anemia and diarrhoea in the previous month were found to have significant association with wasting among HIV-infected children in sub-Saharan Africa (Table 3, supplementary file three).

Three primary articles reported anemia as a factor for wasting among HIV-infected children with a total of 3828 samples [43, 51, 52]. The odds of wasting among anemic HIV positive children were 2.86 times higher than among non-anemic HIV positive children [OR = 2.860 (95% CI: 1.636, 5.000), I^2^ = 74.8%, *P* = 0.019] (Table 3, supplementary file three).

Diarrhoea in the previous month was identified as a factor associated with wasting in three primary studies included in this review [6, 33, 52]. To see the association between Diarrhoea and wasting, 1009 study participants were included in the analysis. Consequently, children who had diarrhoea in the previous month had 4.1 times more odds of wasting than children with no diarrhoea in the previous month [OR = 4.117 (95% CI: 2.876, 5.894), I^2^ = 0.0%, *P* = 0.386] (Table 3, supplementary file three).

## Discussion

Most HIV infected children have an episode of severe malnutrition as their first AIDS-defining illness. Under-nutrition is an important factor which might predict disease progression of HIV-infected individuals. It also results in higher risk of morbidity and mortality in both HIV-infected adults and children. This review was conducted to show the pooled prevalence and associated factors of under-nutrition (stunting, under-weight, and wasting) among HIV infected children in sub-Saharan African countries. This is the first systematic review and meta-analysis on under-nutrition (stunting, under-weight and wasting) among HIV infected children in sub-Saharan African region.

The results of this meta-analysis showed that the pooled prevalence of stunting was 46.7% (95% CI; 40.36–53.07, I^2^ = 98.7%, *p* < 0.01) among HIV infected children in sub-Saharan Africa. This finding was in line with a study conducted in India (46.37%) [62] and meta-analysis conducted in east Africa (49.68%) [63]. However, it was low compared to a study conducted in south India (58%) [64]. The discrepancy might be due to the difference in the number of study participants used by studies. It was higher than the large-scale study conducted among HIV infected adolescents (41%) in the less developed region of the world [65]. The finding was also higher than the WHO estimate of stunting (32.5%) in children regardless of HIV status in African [3]. This is expected since under-nutrition is more prevalent in HIV infected children than uninfected children [8].

The pooled prevalence of underweight was 35.9% (95% CI; 30.79–41.02, I^2^ = 97.4% *p* < 0.01), in this meta-analysis. This finding was lower than a systematic review and meta-analysis study in east Africa (41.63) [63]. It was also lower compared to studies conducted in south India (65%) [64] and India (55.2%) [62]. The discrepancy might be due to the background rate of HIV infection and under-nutrition in the area. In this meta-analysis, the pooled prevalence of wasting was 23.0% (95% CI; 18.67–27.42, I^2^ = 96.9%, *p* < 0.01). Almost similar report was found in systematic review and meta-analysis study conducted in east Africa [63] . This result was higher than studies conducted in south India (16%) [64], in the less developed region of the world (14.5%) [65] and WHO estimate of wasting (6.4%) in Africa [3]. However, it was lower compared to the study finding in India (34.3%) [62]. The reason for the discrepancy may be the difference in sample size and study population.

Regarding factors associated with under-nutrition, advanced WHO HIV/AIDS clinical staging and household food insecurity were significantly associated with stunting among HIV infected children. Family economic status and feeding frequency were found to be significantly associated with underweight. Anemia and diarrhoea in the previous month were also significantly associated with wasting among HIV infected children in sub-Saharan Africa.

The odd of stunting among children with advanced WHO HIV/AIDS clinical staging was 6.7 times higher than their counterparts. Advanced AIDS disease reduces the food appetite of the child due to opportunistic infection that leads to under-nutrition. Children living in food-insecure households were 5.9 times higher odds of stunting than children living in food-secure households. The reason might be that there may be chronic starvation in food-insecure households that easily lead to stunting.

Children whose families had low economic status were 4.7 times more likely to be underweight compared to their counterparts. The reason might be that children who have low family economic status may face poor food access and a lack of a balanced diet results under-weight. HIV infected children who feed 4 times or more per day had 67.8% less odds of under-weight than children feeding less than 4 times per 24 h. This might be due to that low frequency and diversity of feeding demonstrate poor access to food and low micronutrient intake which lead to under-weight [66].

The odds of wasting among HIV positive children who had anemia were 2.9 times higher than among non-anemic HIV positive children. The reason might be that a decrease in the supply of nitrate to the tissue as a result of a decreased blood supply results the child become wasting. Children who had diarrhoea in the previous month had 4.1 times more odds of wasting than children who did not have diarrhoea in the previous month. This might be due to that mal-absorption of nitrate related to frequent loss of stool leading to wasting.

### Limitation of the study

Most primary studies included in this systematic review and meta-analysis were cross-sectional studies which difficulty to established cause-effect relation-ships. The other limitation of this study is the presence of significant heterogeneity between the primary studies and did not consider articles published other than English language.

## Conclusion

This review showed that the prevalence of under- nutrition among HIV infected children was high. Almost half of the HIV infected children became stunted and more than 20% had wasted. The review also showed that two out of five HIV infected children were underweight. Advanced WHO HIV/AIDS clinical staging and household food insecurity were associated with the occurrence of stunting. Low family economic status and low feeding frequency were also associated with under-weight among HIV infected children. Furthermore, anemia and diarrhoea in the previous month were significantly associated with wasting among HIV infected children in sub-Saharan Africa. Nutritional assessment and interventions should give great emphasis during HIV care of children in the ART clinic.

## Supplementary Information

**Additional file 1.**

**Additional file 2.**

**Additional file 3.**

**Additional file 4.**

**Additional file 5.**

## Data Availability

The data used for this study are available and can be accessed from the corresponding author using ‘jemberu2123@gmail.com‘with reasonable request.

## Abbreviations

AIDS: Acquired Immune Deficiency Syndrome
ART: Anti-Retroviral Therapy
HIV: Human Immunodeficiency Virus
WHO: World Health Organizations
OR: odds ratio
CI: confidence interval
WAZ: weight for age z score
WHZ: weight for height z score
HAZ: height for age z score
