# Supplementary material for: Under-nutrition and associated factors among children infected with human immunodeficiency virus in sub-Saharan Africa: a systematic review and meta-analysis
Source: Arch Public Health. 2022 Jan 5;80:19. doi: 10.1186/s13690-021-00785-z (PMC8728950; doi:10.1186/s13690-021-00785-z)
Supplement: Supplementary file 1 — Additional file 1. [file 13690_2021_785_MOESM1_ESM.docx]

**Table 1:** Quality score based on Newcastle-Ottawa Scale of studies included in the systematic review and meta-analysis of under-nutrition and associated factors among HIV infected children in sub-Saharan Africa, 2020.

| Author | publication year | Sample size | stunting | Under-weight | wasting | Quality score | | | |
| --- | --- | --- | --- | --- | --- | --- | --- | --- | --- |
|  |  |  |  |  |  | Selection (from 5 point) | Comparability (from 2 point) | Outcome (from 3 point) | Overall score (from 10 point) |
| Kusum Lata et al | 2020 | 420 | 60.20 | 41.20 | 21.40 | 4 | 2 | 3 | 9 |
| Sunguya et al | 2011 | 213 | 36.60 | 22.10 | 13.60 | 4 | 1 | 3 | 8 |
| Henry Chineme et al | 2014 | 70 | 48.60 | 58.60 | 31.40 | 3 | 2 | 3 | 8 |
| Maura Pedrini et al | 2015 | 140 | 57.40 | 47.10 | 18.60 | 5 | 2 | 2 | 9 |
| Jesson et al | 2015 | 1350 | 32.90 | 36.00 | 16.50 | 4 | 1 | 3 | 9 |
| Megabiaw et al | 2012 | 301 | 65.00 | 41.70 | 5.80 | 4 | 1 | 2 | 8 |
| Poda et al | 2017 | 164 | 29.90 | 11.60 | 10.40 | 3 | 2 | 2 | 7 |
| Calixte Ida Penda et al | 2018 | 217 | 63.60 | 37.80 | 18.40 | 4 | 2 | 2 | 8 |
| Bruno F. Sunguya et al | 2014 | 748 | 61.90 | 26.50 | 6.30 | 5 | 2 | 2 | 9 |
| Andreas Chiabi et al | 2012 | 39 | 51.30 | 56.40 | 20.50 | 3 | 2 | 2 | 7 |
| A.F. Fagbamigbe et al | 2019 | 390 | 36.00 | 50.00 | 50.00 | 4 | 1 | 2 | 7 |
| E. A. anigilaje et al | 2015 | 180 | 54.40 | 12.10 | 33.50 | 3 | 1 | 2 | 6 |
| Teklemariam et al | 2015 | 108 | 49.10 | 51.60 | 31.50 | 3 | 2 | 3 | 8 |
| R. S. Mwiru et al | 2014 | 3144 | 52.00 | 40.00 | 30.00 | 4 | 1 | 2 | 7 |
| Jesson J et al | 2018 | 161 | 52.00 | 52.00 | 36.00 | 5 | 1 | 2 | 8 |
| Cames et al | 2017 | 244 | 42.00 |  | 52.00 | 3 | 2 | 1 | 6 |
| Ute D. Feucht et al | 2016 | 159 | 73.00 | 50.00 | 19.00 | 4 | 1 | 2 | 7 |
| Julie Jesson. et al | 2019 | 3195 | 50.20 | 55.70 | 39.70 | 5 | 2 | 2 | 9 |
| Sofeu CL et al | 2019 | 210 | 77.00 | 53.00 | 47.60 | 4 | 1 | 3 | 8 |
| McHenry MS. et al | 2019 | 426 | 50.90 | 26.50 | 13.60 | 5 | 2 | 2 | 9 |
| Kimani-Murage et al | 2011 | 28 | 28.60 | 10.70 | 7.00 | 3 | 1 | 2 | 6 |
| Sunguya et al | 2012 | 219 | 40.10 | 6.80 | 10.00 | 4 | 2 | 1 | 7 |
| R. Weigel et al | 2010 | 363 | 69.10 | 51.80 |  | 3 | 1 | 2 | 6 |
| Tekleab et al | 2016 | 202 | 71.30 | 39.50 | 16.30 | 3 | 2 | 3 | 8 |
| [David Aguilera et al](https://onlinelibrary.wiley.com/action/doSearch?ContribAuthorStored=Aguilera-Alonso%2C+David) | 2019 | 213 | 56.30 | 56.30 | 27.70 | 4 | 1 | 3 | 8 |
| Julie Jesson et al | 2017 | 308 | 20.00 |  | 31.50 | 3 | 1 | 2 | 6 |
| Asiya et.al. | 2018 | 412 | 13.40 | 21.80 |  | 4 | 1 | 2 | 7 |
| Haileselassie et al | 2019 | 376 | 24.70 |  | 28.20 | 3 | 1 | 3 | 7 |
| Arinaitwe et al | 2012 | 57 | 29.89 | 29.89 |  | 4 | 1 | 1 | 6 |
| Atnafu Mekonnen et al | 2014 | 243 | 62.10 | 15.4 | 2.50 | 4 | 2 | 3 | 9 |
| Kedir et al | 2014 | 560 |  | 51.6 |  | 3 | 1 | 2 | 6 |
| Abdulkadir et al | 2014 | 142 | 46.50 | 40.80 | 31.70 | 4 | 1 | 2 | 7 |
| Arpadi et al | 2019 | 374 | 60.00 | 24.00 | 11.00 | 3 | 2 | 3 | 8 |
| Nalwoga et al | 2010 | 31 | 68.00 | 52.00 | 4.00 | 3 | 1 | 2 | 6 |
