# Supplementary material for: Under-nutrition and associated factors among children infected with human immunodeficiency virus in sub-Saharan Africa: a systematic review and meta-analysis
Source: Arch Public Health. 2022 Jan 5;80:19. doi: 10.1186/s13690-021-00785-z (PMC8728950; doi:10.1186/s13690-021-00785-z)
Supplement: Supplementary file 3 — Additional file 3. [file 13690_2021_785_MOESM3_ESM.docx]

**Figure 1:** Association of WHO HIV/AIDS clinical staging with stunting among HIV infected children in sub-Saharan Africa, 2021.

**Figure 2:** Association of household food insecurity with stunting among HIV infected children in sub-Saharan Africa, 2021.

**Figure 3:** Association of family income with under-weight among HIV infected children in sub-Saharan Africa, 2021.

**Figure 4:** Association of feeding frequency with under-weight among HIV infected children in sub-Saharan Africa, 2021.

**Figure 5:** Association of anemia with wasting among HIV infected children in sub-Saharan Africa, 2021.

**Figure 6:** Association of Diarrhoea with wasting among HIV infected children in sub-Saharan Africa, 2021.
