# Supplementary material for: Under-nutrition and associated factors among children infected with human immunodeficiency virus in sub-Saharan Africa: a systematic review and meta-analysis
Source: Arch Public Health. 2022 Jan 5;80:19. doi: 10.1186/s13690-021-00785-z (PMC8728950; doi:10.1186/s13690-021-00785-z)
Supplement: Supplementary file 4 — Additional file 4. [file 13690_2021_785_MOESM4_ESM.docx]

1. Ahumareze RE, Rankin J, David A, Wapmuk A, Disu E, Balogun Y, Adetunji A, MacArthur E, Amoo O: Prevalence of anaemia and the relationship between haemoglobin concentration and CD4 count in HIV positive children on highly active antiretroviral therapy (HAART) in Lagos, Nigeria. Current Pediatric Research 2016.
2. Nalwoga A, Maher D, Todd J, Karabarinde A, Biraro S, Grosskurth H: Nutritional status of children living in a community with high HIV prevalence in rural Uganda: a cross‐sectional population‐based survey. Tropical medicine & international health 2010, 15(4):414-422.
3. Takarinda KC, Mutasa-Apollo T, Madzima B, Nkomo B, Chigumira A, Banda M, Muti M, Harries AD, Mugurungi O: Malnutrition status and associated factors among HIV-positive patients enrolled in ART clinics in Zimbabwe. BMC Nutrition 2017, 3(1):1-11.
4. Daka DW, Ergiba MS: Prevalence of malnutrition and associated factors among adult patients on antiretroviral therapy follow-up care in Jimma Medical Center, Southwest Ethiopia. PloS one 2020, 15(3):e0229883.
5. Koethe JR, Chi BH, Megazzini KM, Heimburger DC, Stringer JS: Macronutrient supplementation for malnourished HIV-infected adults: a review of the evidence in resource-adequate and resource-constrained settings. Clinical Infectious Diseases 2009, 49(5):787-798.
6. Musoke PM, Fergusson P: Severe malnutrition and metabolic complications of HIV-infected children in the antiretroviral era: clinical care and management in resource-limited settings. The American journal of clinical nutrition 2011, 94(6):1716S-1720S.
7. Osterbauer B, Kapisi J, Bigira V, Mwangwa F, Kinara S, Kamya MR, Dorsey G: Factors associated with malaria parasitaemia, malnutrition, and anaemia among HIV-exposed and unexposed Ugandan infants: a cross-sectional survey. Malaria journal 2012, 11(1):1-6.
8. Abdulahi A, Shab-Bidar S, Rezaei S, Djafarian K: Nutritional status of under five children in Ethiopia: a systematic review and meta-analysis. Ethiopian journal of health sciences 2017, 27(2):175-188.
9. Abate BB, Aragie TG, Tesfaw G: Magnitude of underweight, wasting and stunting among HIV positive children in East Africa: A systematic review and meta-analysis. PloS one 2020, 15(9):e0238403.
10. Ivers LC, Cullen KA, Freedberg KA, Block S, Coates J, Webb P, Mayer KH: HIV/AIDS, undernutrition, and food insecurity. Clinical Infectious Diseases 2009, 49(7):1096-1102.
11. Matara F, Mukona D, Zvinavashe M: Factors contributing to malnutrition among HIV positive children aged between 6 and 60 months. J Nurs Health Sci 2015, 4(1):2320.
12. Muenchhoff M, Healy M, Singh R, Roider J, Groll A, Kindra C, Sibaya T, Moonsamy A, McGregor C, Phan MQ: Malnutrition in HIV-infected children is an indicator of severe disease with an impaired response to antiretroviral therapy. AIDS research and human retroviruses 2018, 34(1):46-55.
13. Jesson J, Schomaker M, Malasteste K, Wati DK, Kariminia A, Sylla M, Kouadio K, Sawry S, Mubiana‐Mbewe M, Ayaya S: Stunting and growth velocity of adolescents with perinatally acquired HIV: differential evolution for males and females. A multiregional analysis from the IeDEA global paediatric collaboration. Journal of the International AIDS Society 2019, 22(11):e25412.
14. Shet A, Mehta S, Rajagopalan N, Dinakar C, Ramesh E, Samuel N, Indumathi C, Fawzi WW, Kurpad AV: Anemia and growth failure among HIV-infected children in India: a retrospective analysis. BMC pediatrics 2009, 9(1):1-9.
15. Padmapriyadarsini C, Pooranagangadevi N, Chandrasekaran K, Subramanyan S, Thiruvalluvan C, Bhavani P, Swaminathan S: Prevalence of underweight, stunting, and wasting among children infected with human immunodeficiency virus in South India. International journal of pediatrics 2009, 2009.
16. Obasohan PE, Walters SJ, Jacques R, Khatab K: Risk factors associated with malnutrition among children under-five years in sub-Saharan African countries: A scoping review. International journal of environmental research and public health 2020, 17(23):8782.
17. Kabalimu TK, Sungwa E, Lwabukuna WC: Malnutrition and associated factors among adults starting on antiretroviral therapy at PASADA Hospital in Temeke District, Tanzania. Tanzania Journal of Health Research 2018, 20(2).
18. Sewale Y, Hailu G, Sintayehu M, Moges NA, Alebel A: Magnitude of malnutrition and associated factors among HIV infected children attending HIV-care in three public hospitals in East and West Gojjam Zones, Amhara, Northwest, Ethiopia, 2017: a cross-sectional study. BMC research notes 2018, 11(1):1-6.
19. Alebel A, Wagnew F, Tesema C, Kibret GD, Petrucka P, Eshite S: Effects of undernutrition on survival of human immunodeficiency virus positive children on antiretroviral therapy. Italian journal of pediatrics 2018, 44(1):1-10.
20. Gedle D, Gelaw B, Muluye D, Mesele M: Prevalence of malnutrition and its associated factors among adult people living with HIV/AIDS receiving anti-retroviral therapy at Butajira Hospital, southern Ethiopia. BMC nutrition 2015, 1(1):1-11.
21. Lentoor AG: Nutritional Status of perinatally HIV‑infected children on antiretroviral therapy from a resource‑poor rural South African community. African Journal of Medical and Health Sciences 2018, 17(1):1-6.
22. Jardim‐Botelho A, Brooker S, Geiger SM, Fleming F, Souza Lopes AC, Diemert DJ, Corrêa‐Oliveira R, Bethony JM: Age patterns in undernutrition and helminth infection in a rural area of Brazil: associations with ascariasis and hookworm. Tropical medicine & international health 2008, 13(4):458-467.
23. Rawat R, Kadiyala S, McNamara PE: The impact of food assistance on weight gain and disease progression among HIV-infected individuals accessing AIDS care and treatment services in Uganda. BMC public health 2010, 10(1):1-8.
24. Ivers LC, Chang Y, Jerome JG, Freedberg KA: Food assistance is associated with improved body mass index, food security and attendance at clinic in an HIV program in central Haiti: a prospective observational cohort study. AIDS research and therapy 2010, 7(1):1-8.
25. Friis H: Micronutrients and HIV infection: a review of current evidence: Consultation on Nutrition and HIV/AIDS in Africa: Evidence, lessons and recommendations for action. Durban, South Africa: Department of Nutrition for Health and Development World Health Organization 2005.
26. Hadgu TH, Worku W, Tetemke D, Berhe H: Undernutrition among HIV positive women in Humera hospital, Tigray, Ethiopia, 2013: antiretroviral therapy alone is not enough, cross sectional study. BMC public health 2013, 13(1):1-10.
27. Hailemariam S, Bune GT, Ayele HT: Malnutrition: Prevalence and its associated factors in People living with HIV/AIDS, in Dilla University Referral Hospital. Archives of Public Health 2013, 71(1):1-11.
28. Hu W, Jiang H, Chen W, He S-H, Deng B, Wang W-Y, Wang Y, Lu CD, Klassen K, Zeng J: Malnutrition in hospitalized people living with HIV/AIDS: evidence from a cross-sectional study from Chengdu, China. Asia Pacific Journal of Clinical Nutrition 2011, 20(4):544-550.
29. Andrade CS, Jesus RP, Andrade TB, Oliveira NS, Nabity SA, Ribeiro GS: Prevalence and characteristics associated with malnutrition at hospitalization among patients with acquired immunodeficiency syndrome in Brazil. PloS one 2012, 7(11):e48717.
30. Daniel M, Mazengia F, Birhanu D: Nutritional status and associated factors among adult HIV/AIDS clients in Felege Hiwot Referral Hospital, Bahir Dar, Ethiopia. Science Journal of Public Health 2013, 1(1):24-31.
31. Kim RJ, Rutstein RM: Impact of antiretroviral therapy on growth, body composition and metabolism in pediatric HIV patients. Pediatric Drugs 2010, 12(3):187-199.
32. Zemede Z, Tariku B, Kote M, Estifanos W: Undernutrition and associated factors among HIV-positive adult patients enrolled in antiretroviral therapy (ART) clinics in the Arba Minch area, southern Ethiopia. HIV/AIDS (Auckland, NZ) 2019, 11:147.
33. Paton NI, Sangeetha S, Earnest A, Bellamy R: The impact of malnutrition on survival and the CD4 count response in HIV‐infected patients starting antiretroviral therapy. HIV medicine 2006, 7(5):323-330.
34. Haile A, Hailu M, Tesfaye E: Prevalence and associated factors of malnutrition among adult hospitalized patients at Amhara National Regional State Referral Hospitals, Ethiopia. Age (Omaha) 2015, 18(40):181.
35. Berhe N, Tegabu D, Alemayehu M: Effect of nutritional factors on adherence to antiretroviral therapy among HIV-infected adults: a case control study in Northern Ethiopia. BMC infectious diseases 2013, 13(1):1-9.
36. Thapa R, Amatya A, Pahari DP, Bam K, Newman MS: Nutritional status and its association with quality of life among people living with HIV attending public anti-retroviral therapy sites of Kathmandu Valley, Nepal. AIDS research and therapy 2015, 12(1):1-10.
37. Ritte S, Kessy A: Social factors and lifestyle attributes associated with nutritional status of people living with HIV/AIDS attending care and treatment clinics in Ilala district, Dar es Salaam. East African journal of public health 2012, 9(1):33-38.
38. Gebremichael DY, Hadush KT, Kebede EM, Zegeye RT: Food insecurity, nutritional status, and factors associated with malnutrition among people living with HIV/AIDS attending antiretroviral therapy at public health facilities in West Shewa Zone, Central Ethiopia. BioMed research international 2018, 2018.
39. Benzekri NA, Sambou J, Diaw B, Sall EHI, Sall F, Niang A, Ba S, Ngom Guèye NF, Diallo MB, Hawes SE: High prevalence of severe food insecurity and malnutrition among HIV-infected adults in Senegal, West Africa. PloS one 2015, 10(11):e0141819.
40. Mitiku A, Ayele TA, Assefa M, Tariku A: Undernutrition and associated factors among adults living with Human Immune Deficiency Virus in Dembia District, northwest Ethiopia: an institution based cross-sectional study. Archives of Public Health 2016, 74(1):1-8.
41. Dedha M, Damena M, Egata G, Negesa L: Undernutrition and associated factors among adults human immunodeficiency virus positive on antiretroviral therapy in hospitals, East Hararge Zone, Oromia, Ethiopia: A cross-sectional study. International journal of health sciences 2017, 11(5):35.
42. Uthman OA: Prevalence and pattern of HIV-related malnutrition among women in sub-Saharan Africa: a meta-analysis of demographic health surveys. BMC public health 2008, 8(1):1-8.
43. Argemi X, Dara S, You S, Mattei JF, Courpotin C, Simon B, Hansmann Y, Christmann D, Lefebvre N: Impact of malnutrition and social determinants on survival of HIV-infected adults starting antiretroviral therapy in resource-limited settings. Aids 2012, 26(9):1161-1166.
44. Martín-Cañavate R, Sonego M, Sagrado MJ, Escobar G, Rivas E, Ayala S, Castaneda L, Aparicio P, Custodio E: Dietary patterns and nutritional status of HIV-infected children and adolescents in El Salvador: A cross-sectional study. PloS one 2018, 13(5):e0196380.
45. Khatri S, Amatya A, Shrestha B: Nutritional status and the associated factors among people living with HIV: an evidence from cross-sectional survey in hospital based antiretroviral therapy site in Kathmandu, Nepal. BMC nutrition 2020, 6(1):1-13.
46. Buonomo E, De Luca S, Tembo D, Scarcella P, Germano P, Doro Altan AM, Palombi L, Liotta G, Nielsen-Saines K, Erba F: Nutritional rehabilitation of HIV-exposed infants in Malawi: results from the drug resources enhancement against AIDS and malnutrition program. International journal of environmental research and public health 2012, 9(2):421-434.
47. Magadi MA: Household and community HIV/AIDS status and child malnutrition in sub-Saharan Africa: evidence from the demographic and health surveys. Social science & medicine 2011, 73(3):436-446.
48. Jesson J, Leroy V: Challenges of malnutrition care among HIV-infected children on antiretroviral treatment in Africa. Medecine et maladies infectieuses 2015, 45(5):149-156.
49. Nasir M, Yeo J, Huang M, Azahar M, Koh M, Khor G: Nutritional status of children living with HIV and receiving antiretroviral (ARV) medication in the Klang Valley, Malaysia. Malaysian journal of nutrition 2011, 17(1).
50. Prendergast A, Bwakura-Dangarembizi MF, Cook AD, Bakeera-Kitaka S, Natukunda E, Ntege PN, Nathoo KJ, Karungi C, Lutaakome J, Kekitiinwa A: Hospitalization for severe malnutrition among HIV-infected children starting antiretroviral therapy. Aids 2011, 25(7):951-956.
51. Preidis GA, McCollum ED, Mwansambo C, Kazembe PN, Schutze GE, Kline MW: Pneumonia and malnutrition are highly predictive of mortality among African children hospitalized with human immunodeficiency virus infection or exposure in the era of antiretroviral therapy. The Journal of pediatrics 2011, 159(3):484-489.
52. Rose AM, Hall CS, Martinez-Alier N: Aetiology and management of malnutrition in HIV-positive children. Archives of disease in childhood 2014, 99(6):546-551.
53. Saloojee H, De Maayer T, Garenne ML, Kahn K: What's new? Investigating risk factors for severe childhood malnutrition in a high HIV prevalence South African setting1. Scandinavian Journal of Public Health 2007, 35(69_suppl):96-106.
54. Magadi MA: Cross‐national analysis of the risk factors of child malnutrition among children made vulnerable by HIV/AIDS in sub‐Saharan Africa: evidence from the DHS. Tropical medicine & international health 2011, 16(5):570-578.
55. Mda S: Multi-micronutrient supplementation in HIV-infected South African children: effect on nutritional s tatus, diarrhoea and respiratory infections; 2011.
56. Mduma ER, Gratz J, Patil C, Matson K, Dakay M, Liu S, Pascal J, McQuillin L, Mighay E, Hinken E: The etiology, risk factors, and interactions of enteric infections and malnutrition and the consequences for child health and development study (MAL-ED): description of the Tanzanian site. Clinical Infectious Diseases 2014, 59(suppl_4):S325-S330.
57. Sidemo NB, Hebo SH: Nutritional status and its effect on treatment outcome among HIV-infected children receiving first-line antiretroviral therapy in Arba Minch General Hospital and Arba Minch Health Center, Gamo Zone, Southern Ethiopia: retrospective Cohort Study. In: Nutrition and HIV/AIDS-Implication for Treatment, Prevention and Cure. edn.: IntechOpen; 2019.
58. Slogrove AL, Goetghebuer T, Cotton MF, Singer J, Bettinger JA: Pattern of infectious morbidity in HIV-exposed uninfected infants and children. Frontiers in immunology 2016, 7:164.
59. Sobze MS, Wadoum RG, Temgoua E, Donfack J-H, Ercoli L, Buonomo E, Fokam J, Dongho BD, Onohiol J-F, Zefack Y: Evaluation of the nutritional status of infants from mothers tested positive to HIV/AIDS in the health district of Dschang, Cameroon. The Pan African Medical Journal 2014, 18.
60. Trehan I, Manary MJ: Management of severe acute malnutrition in low-income and middle-income countries. Archives of disease in childhood 2015, 100(3):283-287.
61. Trehan I, O'Hare BA, Phiri A, Heikens GT: Challenges in the management of HIV-infected malnourished children in sub-Saharan Africa. AIDS research and treatment 2012, 2012.
62. Akombi BJ, Agho KE, Hall JJ, Wali N, Renzaho A, Merom D: Stunting, wasting and underweight in sub-Saharan Africa: a systematic review. International journal of environmental research and public health 2017, 14(8):863.
63. Asnakew M: Malnutrition and associated factors among adult individuals receiving highly active antiretroviral therapy in health facilities of Hosanna Town, Southern Ethiopia. Open Access Library Journal 2015, 2(01):1.
64. Audain KA, Zotor FB, Amuna P, Ellahi B: Food supplementation among HIV-infected adults in Sub-Saharan Africa: impact on treatment adherence and weight gain. Proceedings of the Nutrition Society 2015, 74(4):517-525.
65. Bradley SE, Mishra VK: HIV and nutrition among women in sub-Saharan Africa: Macro International Incorporated; 2008.
66. Braitstein P, Ayaya S, Nyandiko WM, Kamanda A, Koech J, Gisore P, Atwoli L, Vreeman RC, Duefield C, Ayuku DO: Nutritional status of orphaned and separated children and adolescents living in community and institutional environments in Uasin Gishu County, Kenya. PLoS One 2013, 8(7):e70054.
67. Chege PM, Ndungu ZW, Gitonga BM: Food security and nutritional status of children under-five in households affected by HIV and AIDS in Kiandutu informal settlement, Kiambu County, Kenya. Journal of Health, Population and Nutrition 2016, 35(1):1-8.
68. Crippina lb: assessment of nutritional and health interventions on HIV infected children under five years in mathare north health centre, nairobi. School of health sciences, kenyatta university; 2011.
69. Ezeonwu B, Ikefuna A, Oguonu T, Okafor H: Prevalence of hematological abnormalities and malnutrition in HIV-infected under five children in Enugu. Nigerian journal of clinical practice 2014, 17(3):303-308.
70. Fergusson P, Tomkins A: HIV prevalence and mortality among children undergoing treatment for severe acute malnutrition in sub-Saharan Africa: a systematic review and meta-analysis. Transactions of the Royal Society of Tropical Medicine and Hygiene 2009, 103(6):541-548.
71. Finkelstein JL, Mehta S, Duggan C: Maternal vitamin D status and child morbidity, anemia, and growth in human immunodeficiency virus-exposed children in Tanzania. The Pediatric infectious disease journal 2012, 31(2).
72. Gedle D, Mekuria G, Kumera G, Eshete T, Feyera F, Ewunetu T: Food insecurity and its associated factors among people living with HIV/AIDS receiving anti-retroviral therapy at Butajira Hospital, Southern Ethiopia. Journal of Nutrition & Food Sciences 2015, 5(2):2-6.
73. Getahun MB, Teshome GS, Fenta FA, Bizuneh AD, Mulu GB, Kebede MA: Determinants of Severe Acute Malnutrition Among HIV-positive Children Receiving HAART in Public Health Institutions of North Wollo Zone, Northeastern Ethiopia: Unmatched Case–Control Study. Pediatric Health, Medicine and Therapeutics 2020, 11:313.
74. Hussen S, Belachew T, Hussien N: Nutritional status and its effect on treatment outcome among HIV infected clients receiving HAART in Ethiopia: a cohort study. AIDS research and therapy 2016, 13(1):1-8.
75. Itaka MB, Omole OB: Prevalence and factors associated with malnutrition among under 5-year-old children hospitalised in three public hospitals in South Africa. African Journal of Primary Health Care and Family Medicine 2020, 12(1):1-7.
76. Kamenju P, Liu E, Hertzmark E, Spiegelman D, Kisenge R, Kupka R, Aboud S, Manji KP, Duggan C, Fawzi WW: Nutritional status and complementary feeding among HIV‐exposed infants: a prospective cohort study. Maternal & child nutrition 2017, 13(3):e12358.
77. Obiako RO, Akase IE, Hassan A, Babadoko A, Musa BO, Balogun Y, Okonkwo L, Yusuf R, Muktar HM, Obadaki M: Malnutrition in HIV: Are patients in early stages of disease and with high CD4 counts spared? Sub-Saharan African Journal of Medicine 2019, 6(1):43.
78. Okechukwu AA, Okechukwu OA, Chiaha IO: Burden of HIV infection in children with severe malnutrition at the University of Abuja Teaching Hospital, Nigeria. Journal of HIV for Clinical and Scientific Research 2015, 2(2):055-061.
79. Félicitée N, Séraphin N, Amamatou L, Njong TN, Roger D: Additional risk factors for malnutrition in children infected with HIV. JMR 2018, 4(2):63-68.
80. Duggal S, Chugh TD, Duggal AK: HIV and malnutrition: effects on immune system. Clinical and developmental immunology 2012, 2012.
81. Koethe JR, Heimburger DC: Nutritional aspects of HIV-associated wasting in sub-Saharan Africa. The American journal of clinical nutrition 2010, 91(4):1138S-1142S.
